# Rewiring of glucose metabolism defines trained immunity induced by oxidized low-density lipoprotein

**DOI:** 10.1007/s00109-020-01915-w

**Published:** 2020-04-30

**Authors:** Samuel T. Keating, Laszlo Groh, Kathrin Thiem, Siroon Bekkering, Yang Li, Vasiliki Matzaraki, Charlotte D. C. C. van der Heijden, Jelmer H. van Puffelen, Ekta Lachmandas, Trees Jansen, Marije Oosting, L. Charlotte J. de Bree, Valerie A. C. M. Koeken, Simone J. C. F. M. Moorlag, Vera P. Mourits, Janna van Diepen, Rinke Strienstra, Boris Novakovic, Hendrik G. Stunnenberg, Reinout van Crevel, Leo A. B. Joosten, Mihai G. Netea, Niels P. Riksen

**Affiliations:** 1 Department of Internal Medicine (463), Radboud University Medical Center, PO Box 9101, 6500 HB Nijmegen, the Netherlands; 2 Department of Genetics, University Medical Center Groningen, Groningen, the Netherlands; 3 Department for Health Evidence, Radboud University Medical Center, Nijmegen, the Netherlands; 4 Research Center for Vitamins and Vaccines, Bandim Health Project, Statens Serum Institut, Copenhagen, Denmark; 5 Odense Patient Data Explorative Network, University of Southern Denmark/Odense University Hospital, Odense, Denmark; 6 Division of Human Nutrition and Health, Wageningen University, 6700 AA Wageningen, the Netherlands; 7 Faculty of Science, Department of Molecular Biology, Radboud University, 6525 GA Nijmegen, the Netherlands; 8 Present Address: Complex Disease Epigenetics, Murdoch Children’s Research Institute and Department of Paediatrics, University of Melbourne, Parkville, VIC 3052 Australia; 9 Department of Medical Genetics, Iuliu Haţieganu University of Medicine and Pharmacy, Cluj-Napoca, Romania; 10 Department for Genomics & Immunoregulation, Life and Medical Sciences Institute (LIMES), University of Bonn, 53115 Bonn, Germany

**Keywords:** Trained immunity, Atherosclerosis, Immunometabolism, Inflammation, Cardiovascular disease, Diabetes complications, Glycolysis

## Abstract

**Abstract:**

Stimulation of monocytes with microbial and non-microbial products, including oxidized low-density lipoprotein (oxLDL), induces a protracted pro-inflammatory, atherogenic phenotype sustained by metabolic and epigenetic reprogramming via a process called *trained immunity*. We investigated the intracellular metabolic mechanisms driving oxLDL-induced trained immunity in human primary monocytes and observed concomitant upregulation of glycolytic activity and oxygen consumption. In two separate cohorts of healthy volunteers, we assessed the impact of genetic variation in glycolytic genes on the training capacity of monocytes and found that variants mapped to glycolytic enzymes *PFKFB3* and *PFKP* influenced trained immunity by oxLDL. Subsequent functional validation with inhibitors of glycolytic metabolism revealed dose-dependent inhibition of trained immunity in vitro. Furthermore, in vivo administration of the glucose metabolism modulator metformin abrogated the ability for human monocytes to mount a trained response to oxLDL. These findings underscore the importance of cellular metabolism for oxLDL-induced trained immunity and highlight potential immunomodulatory strategies for clinical management of atherosclerosis.

**Key messages:**

- Brief stimulation of monocytes to oxLDL induces a prolonged inflammatory phenotype.
- This is due to upregulation of glycolytic metabolism.
- Genetic variation in glycolytic genes modulates oxLDL-induced trained immunity.
- Pharmacological inhibition of glycolysis prevents trained immunity.

**Electronic supplementary material:**

The online version of this article (10.1007/s00109-020-01915-w) contains supplementary material, which is available to authorized users.

## Introduction

Atherosclerosis is characterized by chronic low-grade inflammation of the arterial wall and monocyte-derived macrophages are the most abundant cells in atherosclerotic plaques [1]. We recently established that monocytes and macrophages can build a long-term pro-inflammatory memory following brief exposure to microbial products (e.g., Bacille Calmette-Guérin (BCG), and the fungal cell wall component β-glucan), by a process called *trained immunity* [2, 3]. Importantly, trained immunity is also induced by sterile, endogenous compounds known to contribute to atherosclerosis, such as oxidized low-density lipoprotein (oxLDL), lipoprotein (a), and aldosterone [4–6]. Cells trained with oxLDL are characterized by an increased cytokine production capacity and enhanced foam cell formation [4]. Therefore, while trained immunity is beneficial in the context of host defense against micro-organisms, it may play a maladaptive role in chronic inflammatory diseases [7]. To this end, we recently hypothesized that trained immunity contributes to the persistent inflammation in atherosclerosis [8, 9]. Indeed, circulating monocytes isolated from patients with established atherosclerosis or patients with hypercholesterolemia exhibit a trained phenotype [10, 11].

Trained immunity induced by β-glucan or BCG is associated with profound intracellular metabolic reprogramming, characterized by increased glycolytic metabolism and intracellular accumulation of fumarate and mevalonate [2, 12–14]. β-Glucan training is furthermore accompanied by the repression of oxidative phosphorylation (OXPHOS), reminiscent of Warburg metabolism, whereas BCG-induced trained immunity is supported by concomitant increases in glycolysis and OXPHOS [15].

At the level of gene regulation, trained immunity is characterized by epigenetic changes that modulate transcriptional programs. Studies of cells trained with β-glucan [16] and BCG [15] have associated the enrichment of H3 histones trimethylated at lysine 4 (H3K4me3) at regulatory promoters with increased expression of genes involved in glycolytic metabolism, thus linking immunometabolic and epigenetic reprogramming. On the other hand, there is evidence of a reverse-causal relationship, whereby blocking the activation of aerobic glycolysis precludes the characteristic chromatin modification pattern and adapted phenotype of trained immunity [16].

The current study is aimed at unraveling the role of metabolic reprogramming in oxLDL-induced trained immunity.

## Materials and methods

### Cells and reagents

Buffy coats from healthy donors were obtained after written informed consent (Sanquin Blood Bank, Nijmegen, the Netherlands). Human peripheral blood mononuclear cells (PBMCs) were isolated by density-gradient centrifugation over Ficoll-Paque (GE Healthcare). Percoll isolation of monocytes was performed as previously described as yielding a level of T cell contamination, measured by fluorescence-activated cell sorting of only 5% [13, 17]. Purified monocytes were cultured in RPMI 1640 Dutch-modified culture medium (RPMI medium, Invitrogen) supplemented with 50 μg/mL gentamicin (Centraform), 2 mmol/L Glutamax (Invitrogen), 1 mmol/L pyruvate (Invitrogen), and 10% pooled human serum. Stimuli and inhibitors used were *Escherichia coli* lipopolysaccharide (LPS; serotype 055:B5, Sigma-Aldrich, 10 ng/mL), Pam3Cys (EMC Microcollections, L2000, 10 μg/mL), 3-(3-pyridinyl)-1-(4-pyridinyl)-2-propen-1-one (3PO, Sigma-Aldrich), and 2-deoxy-d-glucose (2-DG, Sigma-Aldrich). Low-density lipoprotein was isolated from pooled human serum by ultracentrifugation and oxidized by incubating with 20 μmol CuSO_4_/L for 16 h at 37 °C followed by dialysis, as previously described [4].

### In vitro training and pharmacological inhibition

Adherent monocytes were trained as described previously [17]. Briefly, cells were incubated with oxLDL (10 μg/mL) for 24 h, washed with phosphate-buffered saline (PBS), and incubated in normal culture medium at 37 °C, 5% CO_2_. For pharmacological inhibition experiments, cells were co-incubated with inhibitors (3PO [10–40 μmol/L], 2-DG [1 mmol/L], metformin [10 μmol/L]) for the 24 h of oxLDL stimulation. For glucose experiments, cells were incubated with oxLDL (10 μg/mL) in culture medium supplemented with 5 mM glucose (+ 20 mM mannitol) or 25 mM glucose for 24 h, washed with warm PBS, and incubated with RPMI supplemented with 6 mM glucose and 10% pooled human serum (obtained anonymously from the laboratory of our hospital) at 37 °C, 5% CO_2_. Following 5 days in culture, cells were restimulated with medium alone, 10 ng/mL LPS.

### Cytokine measurement

Cytokine production in supernatants after 24 h or 7 days was determined using commercial enzyme-linked immunosorbent assay kits for TNF-α and IL-6 (R&D Systems, MN, USA) according to the instructions of the manufacturers.

### Quantitative RT-PCR

Total RNA was isolated from macrophages using TRIzol reagent according to the manufacturer’s instructions. 0.5–1 μg of total RNA was used to synthesize cDNA with the SuperScript III First-Strand Synthesis System (Thermo Fisher Scientific) according to the manufacturer’s protocol. Quantitative RT-PCR was performed using an Applied Biosciences StepOnePLUS qRT-PCR machine using SYBR Green (Invitrogen). All reactions were performed for at least 6 biological replicates and the values expressed as log2 fold increase in mRNA levels relative to those in non-trained cells. *18s* was used as a housekeeping gene. RT-PCR primers are listed in Table S1**.**

### Chromatin immunoprecipitation

Trained monocytes on day 6 were cross-linked in methanol-free 1% formaldehyde, followed by sonication and immunoprecipitation using antibodies against H3K4me3 (Diagenode, Seraing, Belgium). Immunoprecipitated chromatin was processed further for qRT-PCR analysis using the MinElute DNA Purification Kit (Qiagen). Primers used in the reaction are listed in Table S1. Samples were analyzed with a comparative Ct method on the StepOnePLUS qPCR machine (Applied Biosystems) using SYBR green (Invitrogen) in accordance with the manufacturer’s instructions.

### Metabolic analysis

Approximately 1 × 10^7^ monocytes were trained with oxLDL (10 μg/mL) in 10-cm Petri dishes (Greiner) in 10 mL medium volumes for 24 h, washed with warm PBS, and incubated in normal culture medium at 37 °C, 5% CO_2_. Following 5 days in culture, cells were detached with Versene solution (Thermo Fisher Scientific) and 1 × 10^5^ cells were plated in quintuplicate to overnight-calibrated cartridges in assay medium (RPMI with 0.6 mmol/L glutamine, 5 mmol/L glucose, and 1 mmol/L pyruvate [pH adjusted to 7.4]) and incubated for 1 h in a non-CO_2_-corrected incubator at 37 °C. Trained and untrained macrophages for each respective donor were included in the same assay. Oxygen consumption rate (OCR) and extracellular acidification rate (ECAR) were measured using a Cell Mito Stress Kit (for OCR) or a glycolysis stress test (for ECAR) kit in an XFp Analyzer (Seahorse Bioscience), with final concentrations of 1 μmol/L oligomycin, 1 μmol/L FCCP, and 0.5 μmol/L rotenone/antimycin A.

### Genetic analysis

We conducted in vitro oxLDL training of adherent PBMCs from 119 healthy individuals of Western European ancestry from the 200 Functional Genomics cohort (2011/399) of the Human Functional Genomics Project (www.humanfunctionalgenomics.org). Genotype information on approximately 8 million single-nucleotide polymorphisms (SNPs) was obtained using Illumina HumanOmniExpressExome SNP chip upon imputation. Only SNPs with a minor allele frequency of ≥ 5% that passed standard quality filters were included in the analysis. Raw cytokine levels were log-transformed and the ratio between trained and non-trained cytokine levels was used to quantify the trained immunity response. They were subsequently mapped to genotype data using a linear regression model with age and gender as covariates [18].

We also conducted in vitro oxLDL training of adherent PBMCs in a second cohort of 243 healthy individuals of Western European ancestry from the 300BCG cohort (NL58553.091.16). DNA samples of the individuals from the second cohort were genotyped using the commercially available SNP chip, Infinium Global Screening Array MD v1.0 from Illumina. Genotype information on approximately 4 million SNPs was obtained upon imputation (MAF > 5% and *R*^2^ > 0.3 for imputation quality).

After removing genetic outliers, cytokine ratios were mapped to genotype data as described for the 200FG cohort.

Ethical approval of the cohort studies was granted by the local Ethics Committee (CMO regio Arnhem-Nijmegen; numbers 2011/399 and NL58553.091.16). Inclusion of volunteers and experiments were conducted according to the principles expressed in the Declaration of Helsinki. All volunteers gave written informed consent before any material was taken.

### Metformin trial

In this prospective study, 11 healthy non-obese volunteers received increasing doses of metformin for a total of 6 days (starting at 500 mg once per day and ending with 1000 mg twice per day). Baseline characteristics of the participants are described in Table S2. Blood was drawn immediately before metformin administration (day 0), immediately after administration (day 6), as well as 3 days (day 9) and 2 weeks (day 20) after the final dose. The study was approved by the local institutional review committee (Arnhem-Nijmegen Medical Ethical Committee, NL47793.091.14) and conducted according to the principles of the International Conference on Harmonization-Good Clinical Practice guidelines. All volunteers gave written informed consent before participation.

### Statistical analysis

Ex vivo and in vitro monocyte experiments were analyzed using a Wilcoxon signed-rank test. R-package Matrix eQTL was used for cytokine QTL mapping. A *p* value < 0.05 (*) was considered statistically significant, (**) *p* < 0.01. Data represent mean ± SEM.

## Results

### oxLDL-induced trained immunity is associated with increased glycolytic metabolism and oxygen consumption

**Fig. 1:**
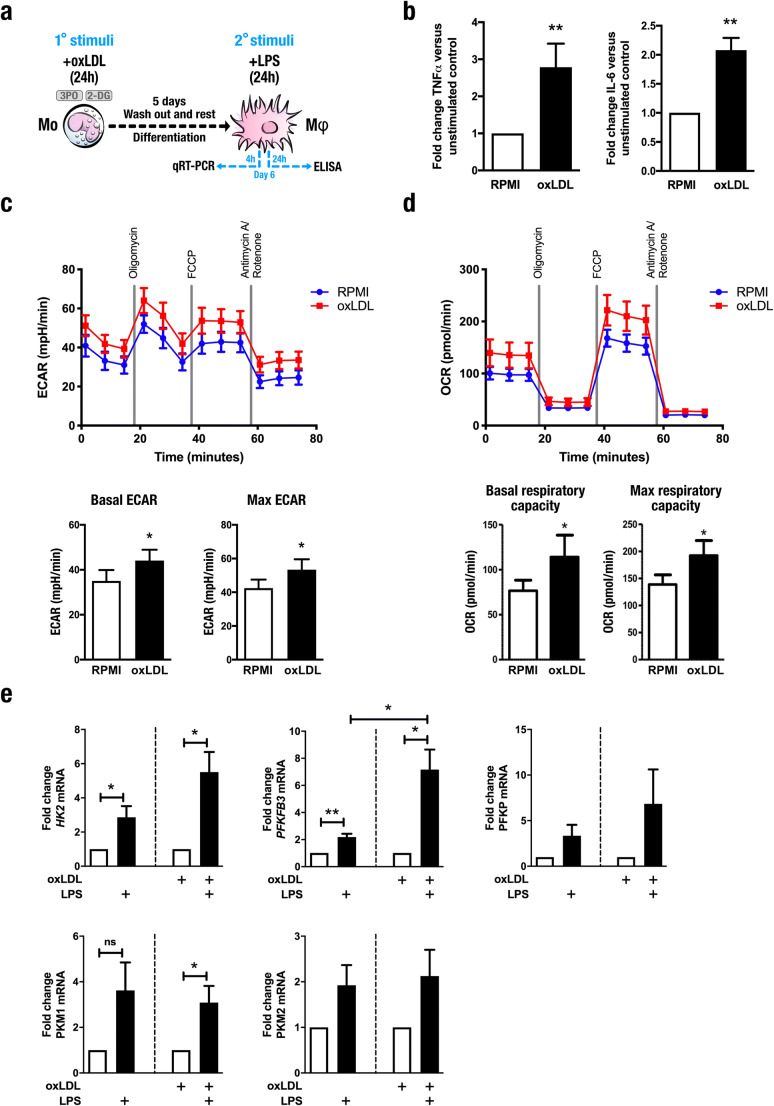
Induction of trained immunity by oxidized low-density lipoprotein is dependent on glycolytic metabolism. **a** Graphical outline of in vitro training methods. **b** Human primary monocytes were incubated for 24 h with culture medium (RPMI, open bars) or oxLDL (closed bars), allowed to rest for 5 days and then stimulated for 24 h with LPS (10 ng/mL), and levels of IL-6 and TNF-α were assessed in supernatants by enzyme-linked immunosorbent assay (mean ± SEM, *n* = 8, **p < 0.01, Wilcoxon signed-rank test). **c** Extracellular acidification rate (ECAR) and **d** oxygen consumption rate (OCR) of cells incubated with RPMI or trained with oxLDL determined by Seahorse XF technology at day 6 (prior to restimulation) (mean ± SEM, *n* = 7, *p < 0.05, Wilcoxon signed-rank test). **e** mRNA expression of genes encoding enzymes involved in glycolysis measured by qRT-PCR 4 h after restimulation with LPS (mean ± SEM, *n* = 6)

To investigate the metabolic phenotype of monocytes trained with oxLDL, we incubated human primary monocytes with culture medium or oxLDL (10 μg/mL) for 24 h. On day 6, the cells were restimulated with culture medium alone or the Toll-like receptor 4 ligand lipopolysaccharide (LPS, 10 ng/mL) for 24 h, after which pro-inflammatory cytokine production was measured (Fig. 1a). Tumor necrosis factor alpha (TNF-α) and interleukin 6 (IL-6) were measured as numerous studies of trained immunity demonstrate that these cytokines are reliable functional readouts for pro-inflammatory cytokine production in trained cells [13, 16]. In accordance with previous findings [4], we observed that cells that had been stimulated with oxLDL exhibited enhanced TNF-α and IL-6 production following LPS restimulation (Fig. 1b).

To understand the mechanisms supporting this phenotype, we analyzed the metabolism of trained cells using Seahorse XF technology. Five days after removal of the oxLDL from the culture medium, trained macrophages were distinguishable by an increased rate of extracellular acidification (ECAR) (Fig. 1c), signifying enhanced glycolytic flux. In addition, the rate of oxygen consumption (OCR) was increased in cells trained with oxLDL (Fig. 1d), indicating concurrent upregulation of OXPHOS. Previous studies have reported transcriptional activation of genes related to glycolysis in trained immunity [10, 15, 16]. To determine if this was also true for macrophages trained with oxLDL, we analyzed the mRNA expression of key glycolytic enzymes. Following 4 h of LPS restimulation (Fig. 1a), we observed a trend toward upregulation of genes encoding glycolytic pathway enzymes *PFKP*, *PKM1*, and *PKM2* in cells incubated with RPMI. Statistically significant differences were observed for *HK2* and *PFKFB3* in untrained macrophages stimulated with LPS. Training with oxLDL exacerbated this effect, particularly for *PFKFB3* mRNA expression. In contrast, the expression of genes encoding pyruvate kinase enzymes *PKM1* and *PKM2* was not significantly altered by oxLDL training (Fig. 1e).

### Genetic variation in glycolytic enzymes determine the individual susceptibility to oxLDL-induced training

**Fig. 2:**
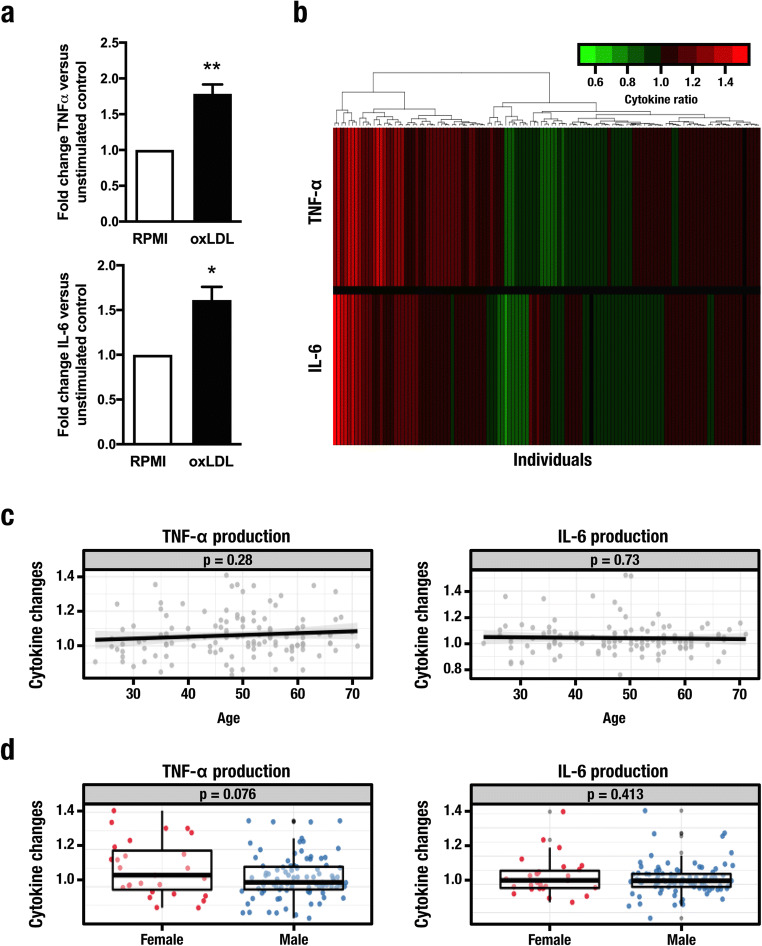
Inter-individual variation in trained immunity mediated by oxidized low-density lipoprotein. Human PBMCs isolated from 119 healthy individuals exposed for 24 h to either culture medium alone or oxidized low-density lipoprotein were maintained in normal culture conditions for 5 days. On day 6, the cells were restimulated with culture medium or lipopolysaccharide (10 ng/mL) for 24 h and production of TNF-α and IL-6 was measured by enzyme-linked immunosorbent assay. **a** Ratios of cytokine production in trained vs. non-trained monocytes isolated from healthy volunteers (Mean ± SEM, *n* = 119, *p < 0.05, **p < 0.01, Student’s *t* test). **b** Fold changes of cytokine levels, with green/red corresponding to decreased/increased cytokine production respectively. **c**, **d** Impact of age (**c**) and gender (**d**) on trained cytokine responses

To further understand the role of the metabolic adaptations, we investigated if genetic variation affects individual susceptibility for trained immunity using a genetic study of 119 healthy volunteers. PBMCs were incubated with culture medium or 10 μg/mL oxLDL for 24 h. Cells were washed and incubated in normal culture conditions for a further 5 days. On day 6, the cells were restimulated with culture medium alone or LPS (10 ng/mL) for 24 h. We confirmed that the oxLDL-dependent augmented production of TNF-α and IL-6 previously seen in enriched monocyte fractions was also detectable in PBMCs **(**Fig. 2a**)**. Furthermore, we observed considerable inter-individual variation in cytokine production by oxLDL-trained cells (Fig. 2b**)**. To investigate the sources of this variation, we explored the potential influence of factors known to affect cytokine production and observed no effect of age (Fig. 2c) or sex (Fig. 2d).

**Fig. 3:**
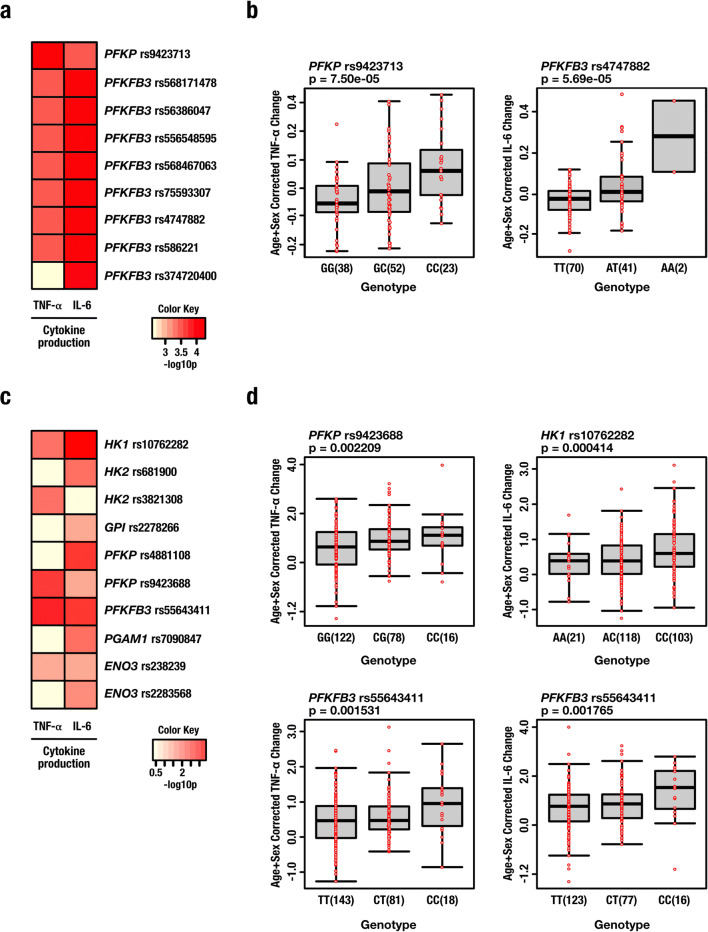
Genetic variation in glycolytic enzymes modifies the training response to oxidized low-density lipoprotein. **a** Single-nucleotide polymorphisms (SNPs) in genes encoding key glycolytic enzymes PFKP and PFKFB3 suggestively associated (*p* < 9.99 × 10^−3^) with trained responses to oxidized low-density lipoprotein (oxLDL) (cohort 1, *n* = 119). **b** Specific SNPs proximal to *PFKP* and *PFKFB3* associated with TNF-α and IL-6 production respectively in cells trained with oxLDL. **c** SNPs in genes encoding key glycolytic enzymes suggestively associated with trained responses to oxidized low-density lipoprotein (oxLDL) (cohort 2, *n* = 215 for TNF-α, *n* = 228 for IL-6). **d** Specific SNPs within *PFKP*, *PFKFB3*, and *HK1* associated with differential pro-inflammatory cytokine production in cells trained with oxLDL

To understand the impact of genetic variation on cytokine production, we tested for associations among SNPs and the magnitude of oxLDL-trained cytokine responses of individual subjects. Genome-wide significant (*p* < 5 × 10^−8^) QTLs were not observed. However, to increase sensitivity, we studied all SNPs with a *p* value < 9.99 × 10^−3^ Using this approach, we identified several SNPs suggestively associated with adaptive changes in cytokine production mapped within 250 kB of genes encoding key glycolytic enzymes. Specifically, genetic variation in genes encoding the inducible PFK-2/FBPase isozyme 6-phosphofructo-2-kinase/fructose-2,6-biphosphatase 3 (PFKFB3) and phosphofructokinase (PFKP) were associated with the potentiation of TNF-α and IL-6 production upon training with oxLDL (Fig. 3a). With regard to TNF-α production by oxLDL-trained cells, the most strongly associated SNP was rs9423713 (*p* = 7.5 × 10^−5^) which is located within an enhancer region approximately 100 kB downstream of *PFKP* (GeneHancer ID: GH10J003171). For IL-6 production, the SNP most strongly associated with oxLDL training was rs4747882 (*PFKFB3*, *p* = 5.69 × 10^−5^) (Fig. 3b). We validated these findings using an independent genetic study of 243 healthy volunteers. Testing for associations among common SNPs (MAF > 5%) and variation in the magnitude of oxLDL-trained cytokine responses across this cohort, we identified several SNPs suggestively associated with adaptive changes in cytokine production mapped within 250 kB of genes associated with glycolysis (*p* < 9.99 × 10^−3^) (Fig. 3c). Like the first cohort, some of the strongest associations were observed for variants mapped to genes encoding *PFKP* and *PFKFB3*. The SNPs most strongly associated to TNF-α production were rs9423688 (intron 16 of *PFKP*, *p* = 0.002) and rs55643411 (exon 15 of *PFKFB3*, *p* = 0.001), whereas SNPs strongly associated with IL-6 production included rs10762282 (intron 4 of *HK1*, *p* = 0.0004) and rs55643411 (*PFKFB3*, *p* = 0.001) (Fig. 3d). Together, these data suggest that glycolysis is a key metabolic pathway for pro-inflammatory cytokine production in trained immunity induced by oxLDL.

### Pharmacological inhibition of glycolysis prevents oxLDL-induced trained immunity

**Fig. 4:**
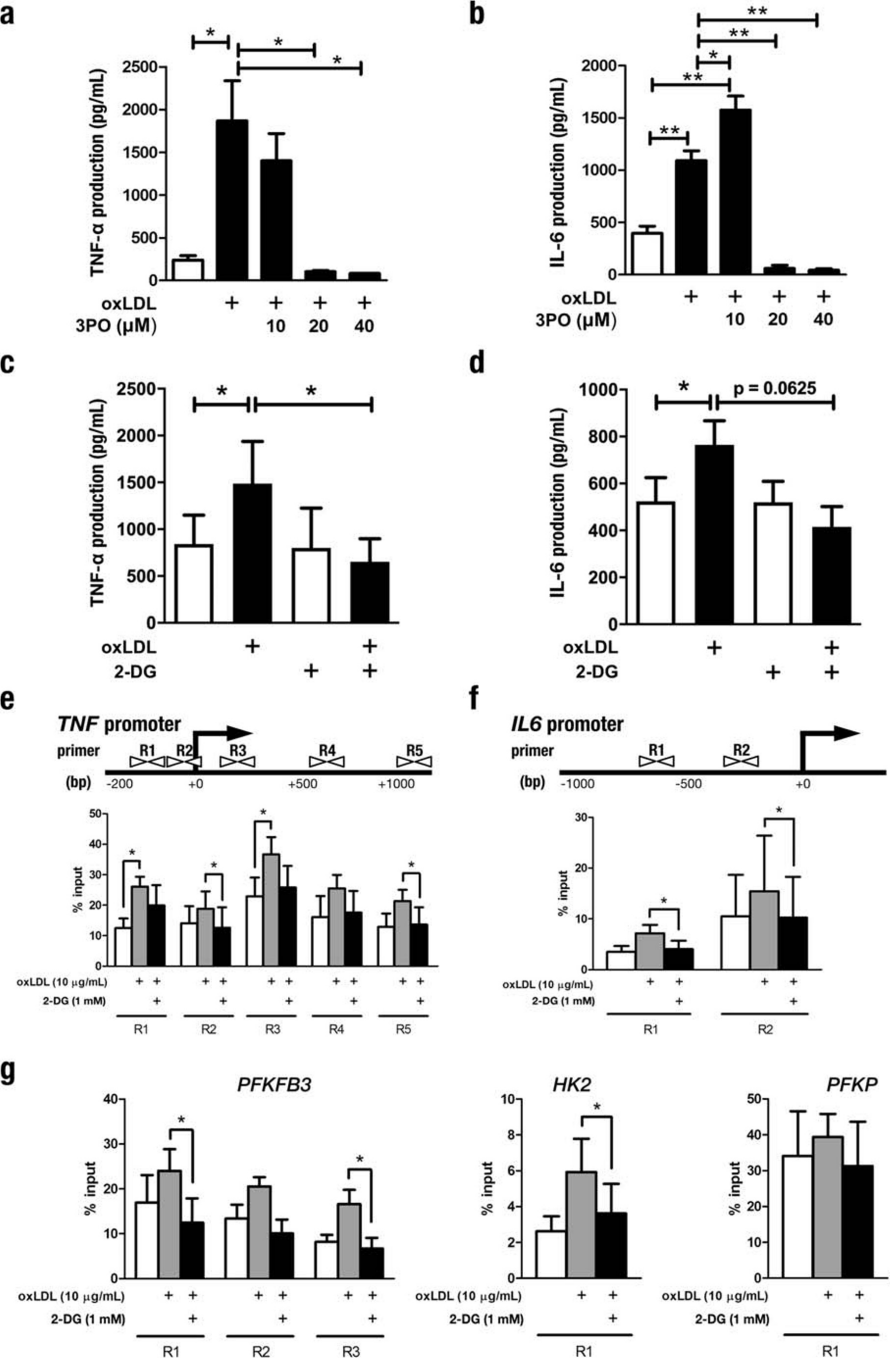
Pharmacological inhibition of glycolysis prevents trained immunity induced by oxLDL. **a**, **b** Monocytes were incubated for 24 h with culture medium (RPMI, open bars) or oxLDL (closed bars), alone or in combination with the specific PFKFB3 inhibitor 3PO. Following restimulation with LPS, TNF-α (**a**) and IL-6 (**b**) production was measured in the supernatants by enzyme-linked immunosorbent assay (mean ± SEM, *n* = 4 *p < 0.05, **p < 0.01, Wilcoxon signed-rank test). **c**, **d** Monocytes were incubated for 24 h with culture medium (RPMI open bars) or oxLDL (closed bars), with or without glycolysis inhibitor 2-DG. At day 6, TNF-α (**c**) and IL-6 (**d**) production was measured by enzyme-linked immunosorbent assay in supernatants after stimulation with LPS (mean ± SEM, *n* = 6 *p < 0.05, **p < 0.01 Wilcoxon signed-rank test). **e**–**g** Soluble chromatin was isolated from cells incubated with RPMI or oxLDL alone or in combination with 2-DG and immunopurified with anti-H3K4me3 antibody. Schematic representations of the human *TNF* and *IL6* genes highlighting the regions (R1–5 for *TNF*, R1–2 for *IL6*) of chromatin specifically analyzed for histone modifications are shown in the top panel of **e** and **f** respectively. Quantitative RT-PCR was used to measure the level of enrichment (lower panels) following co-incubation with oxLDL and 2-DG at promoter regions proximal to genes encoding **e** TNF-α and **f** IL-6. **g** H3K4me3 enrichment was also assessed at the proximal promoters of glycolytic genes *PFKFB3*, *HK2*, and *PFKP* (mean ± SEM, *n* = 6 *p < 0.05, Wilcoxon signed-rank test)

To determine the physiological significance of changes in glycolytic metabolism, we used two distinct approaches to pharmacologically target glycolysis. First, we inhibited PFKFB3, a crucial rate-limiting enzyme in glycolysis, which is upregulated in oxLDL-trained cells [19]. Co-incubation of 3PO with oxLDL for the first 24 h of the in vitro training protocol dose-dependently attenuated the oxLDL-augmented production of TNF-α (Fig. 4a) and IL-6 (Fig. 4b) upon secondary stimulation with LPS. The dependency of oxLDL-mediated trained immunity on glycolytic metabolism was further validated using the direct inhibitor of glycolysis 2-DG, which also blunted the enhanced cytokine production (Fig. 4c and d).

Epigenetic enrichment of transcriptionally permissive H3K4me3 was previously described at the promoters of genes encoding pro-inflammatory cytokines in macrophages trained with oxLDL [4], BCG [15], and β-glucan [16]. We were therefore prompted to investigate the connection between these chromatin patterns and the metabolic reprogramming. We replicated previous reports of H3K4me3 enrichment at regulatory elements immediately upstream of genes encoding TNF-α and IL-6 induced by oxLDL training [4]. Inhibition of glycolysis with 2-DG precluded the enrichment of H3K4me3 at these gene promoters (Fig. 4e and f). Furthermore, we observed similar enrichment of H3K4me3 at the promoters of *PFKFB3* and *HK2* induced by training with oxLDL, which was also attenuated when glycolysis was inhibited by 2-DG. On the other hand, we did not observe H3K4me3 enrichment at the *PFKP* promoter, which is in line with the lack of transcriptional upregulation following LPS exposure (Fig. 4g). Collectively, these data highlight the necessity of glucose metabolism for the induction of trained immunity, and the interconnectedness with epigenetic remodeling.

### High glucose concentrations exacerbate oxLDL-induced trained immunity

**Fig. 5:**
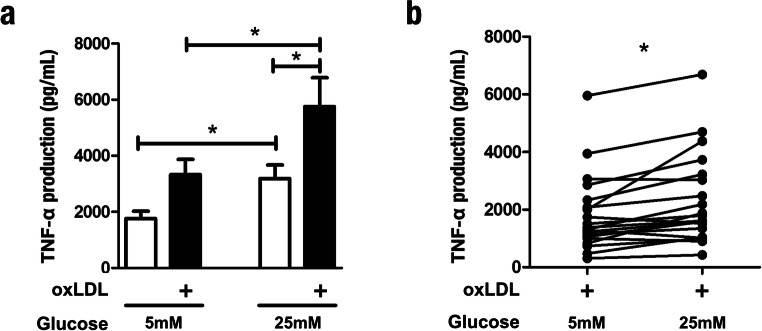
High glucose concentrations exacerbate trained immunity induced by oxLDL. **a** Monocytes were incubated for 24 h with culture medium (open bars) or oxLDL (closed bars) in culture medium supplemented with 25 mM glucose or 5 mM glucose and 20 mM mannitol. Cells were then washed with warm PBS and incubated in normal culture medium supplemented with 6 mM glucose. Following 5 days in culture, the cells were restimulated with medium alone or 10 ng/mL LPS for 24 h and TNF-α production was measured in the supernatants by enzyme-linked immunosorbent assay (mean ± SEM, *n* = 20, *p < 0.05, Student’s *t* test). **b** TNF-α production by cells isolated from the same individual and trained with oxLDL in medium supplemented with 5 mM glucose and 25 mM glucose (*n* = 20, *p < 0.05, Wilcoxon signed-rank test)

Atherosclerotic cardiovascular disease (ASCVD) risk is severely elevated in individuals with diabetes [20]. Moreover, hyperglycemia is an independent risk factor for ASCVD in the diabetic population [9, 21, 22]. We hypothesized that glucose availability could modulate the pro-inflammatory, glycolysis-dependent properties of trained immunity, thereby amplifying the already heightened atherogenicity of macrophages trained with oxLDL. In general, studies of primary monocytes and macrophages, including investigations of trained immunity, are conducted using 11 mM glucose in vitro [17]. We confirmed that human primary monocytes could be trained with oxLDL in cell culture medium supplemented with the more physiologically relevant glucose concentration of 5 mM, thus demonstrating that in vitro trained immunity is not an artifact of specific culture conditions (Fig. 5a). Next, we measured the capacity of monocytes isolated from the same individuals to be trained under variable glucose concentrations. Indeed, 24-h co-incubation with 25 mM glucose exacerbated the training effect of oxLDL on TNF-α production, compared with cells trained in the presence of 5 mM glucose (Fig. 5a). Furthermore, we observed that overnight incubation with a high concentration glucose alone induced a trained phenotype (Fig. 5a, compare open bars). While considerable variation in the magnitude of oxLDL-induced training was observed at 5 mM glucose, the high glucose medium augmented the trained TNF-α production in nearly all individuals tested (Fig. 5b).

### Metformin prevents oxLDL-induced trained immunity in vitro and in vivo

**Fig. 6:**
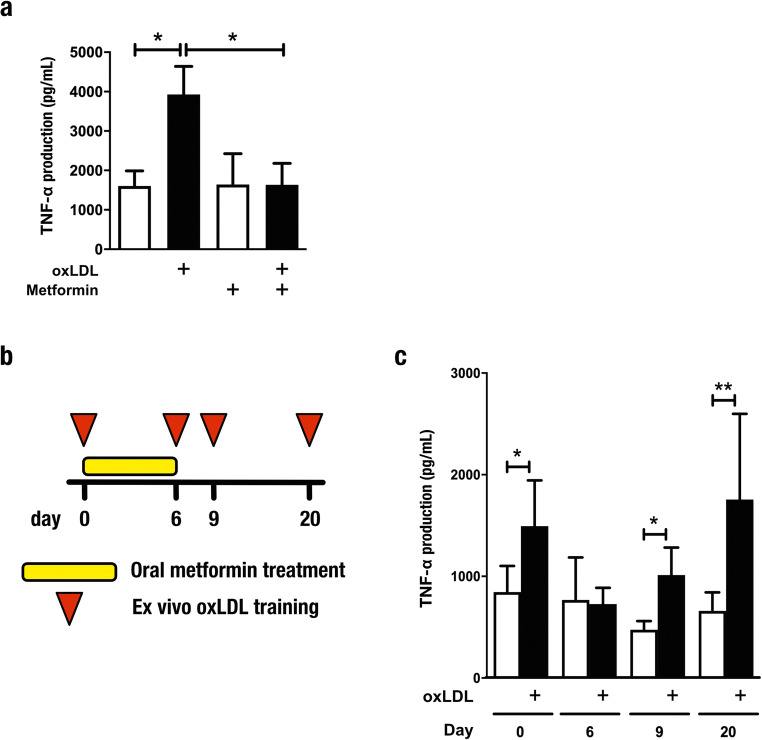
Metformin prevents oxLDL-induced trained immunity in vitro and in vivo. **a**, **b** TNF-α production by human monocytes trained in vitro with oxidized low-density lipoprotein (oxLDL, closed bars) and restimulated with lipopolysaccharide (10 ng/mL) (**a**) in the presence or absence of 10 μM metformin (mean ± SEM, *n* = 5 *p < 0.05, Wilcoxon signed-rank test). **c** Graphical outline of the in vivo metformin study. Peripheral blood mononuclear cells were isolated from healthy volunteers before initiation of treatment, during treatment, and after treatment with metformin, and exposed for 24 h to oxLDL (10 μg/mL). **d** At day 6 of the ex vivo training protocol, the cells were restimulated with lipopolysaccharide and Pam3Cys and cytokines were measured by enzyme-linked immunosorbent assay (mean ± SEM, *n* = 11, *p < 0.05, **p < 0.01 compared with RPMI control, Wilcoxon signed-rank test)

Metformin is the antihyperglycemic drug of first choice in patients with type 2 diabetes mellitus. While the mechanism of action is complex, metformin is known to activate AMP-activated protein kinase and subsequently inhibit mechanistic target of rapamycin (mTOR) [23]. In addition, metformin also inhibits complex I of the mitochondria electron transport chain and thereby inhibits OXPHOS. We previously demonstrated that inhibition of the mTOR pathway by metformin counteracts the induction of trained immunity by β-glucan in vivo [13, 16]. To assess whether metformin can similarly modulate oxLDL-mediated trained immunity, we first investigated the effects in vitro. Following restimulation with LPS, TNF-α production was significantly reduced in cells co-incubated with oxLDL and metformin (Fig. 6a). Next, we conducted a study in healthy volunteers treated with metformin for 6 days. Peripheral blood mononuclear cells were isolated before initiation of treatment, during metformin treatment (6-day time point) and after treatment (9- and 20-day time points), and trained ex vivo for 24 h with oxLDL (Fig. 6b). We observed that oxLDL-induced trained immunity was abolished by metformin in vivo (6-day time point), and restored after cessation of treatment (Fig. 6c).

## Discussion

The ability of human innate immune cells to build a de facto immunological memory of infectious challenges has been recently described [2]. In parallel, we recently showed that exposure of monocytes to a low concentration of oxLDL induces a sustained pro-inflammatory and atherogenic phenotype [4]. This trained immunity phenotype can also be induced by a Western type diet in *Ldlr−/−* mice and likely represents an important novel mechanism in atherogenesis [24]. We now provide several lines of evidence demonstrating that the development of trained immunity by oxLDL is critically dependent on changes in intracellular metabolism, in particular glycolysis and oxidative phosphorylation. These findings improve our understanding of the mechanism of trained immunity induced by oxLDL and point toward novel targets for pharmacotherapy.

The importance of changes in cellular glucose metabolism was previously indicated for the development of atherosclerosis [12]. In atherosclerotic plaque macrophages, glycolytic metabolism is elevated [25]. In accordance with our findings, mRNA expression of glycolytic rate-limiting enzymes hexokinase 2 (*HK2*) and *PFKFB3* is significantly elevated in monocytes isolated from patients with symptomatic atherosclerosis [10]. Furthermore, attenuation of glycolysis by silencing or inhibiting *Pfkfb3* in high fat-fed *ApoE*^−/−^ mice correlated with a significant reduction in aortic tissue levels of TNF-α and CCL2 [26]. This association is further underscored by recent reports describing that glucose over-utilization drives the excessive production of IL-6 and IL-1β by monocytes and macrophages derived from patients with coronary artery disease, by a process that is dependent on redox-sensitive STAT3 signaling [27].

Here, we describe the critical importance of glucose metabolism for monocytes to build an immunological memory after oxLDL stimulation, underwriting their capacity to mount a subsequently heightened response to TLR stimulation. Our results closely mirror aspects of the metabolic reprogramming induced by microbial stimulators of trained immunity. Indeed, genetic variation in *HK2* and *PFKP* modulates the induction of trained immunity by BCG [15]. Furthermore, we demonstrate that this activation of glycolysis is at least partly dependent on the epigenetic modification of promoters of genes that regulate glycolytic metabolism.

By supplying the energy required for different states of activation, metabolic pathways distinguish and support the spectrum of macrophage phenotypes. Though energetically less efficient than OXPHOS, a potential explanation for the dependency on glycolysis is that this metabolic pathway can be rapidly amplified to meet the ATP requirements of trained cells, and that the ATP producing capacity of the TCA cycle is limited by anaplerotic repurposing of TCA cycle intermediates, as described for β-glucan-induced training [13]. Indeed, trained monocytes consume significantly more glucose than naive cells under resting conditions [15, 16]. On the other hand, the metabolic profiles induced by these stimuli are distinguishable by OXPHOS: OXPHOS is increased in cells trained with BCG and oxLDL, whereas cells trained with β-glucan exhibit a marked reduction in oxygen consumption while increasing their glucose utilization [16]. Currently, the underlying mechanisms and broader implications of these differences are unclear. Our study is limited by our focus on the role of glycolysis in oxLDL-mediated metabolic regulation in trained immunity. While this is clearly a crucial pathway for the induction of trained immunity by other stimuli such as BCG [15] and β-glucan [16], inhibition of the electronic transport chain by metformin also leads to downregulation of oxLDL-induced trained immunity, suggesting a role for OXPHOS in the establishment of the maladaptive phenotype by oxLDL.

Our observation that pharmacological blockade of glycolysis prevents oxLDL-mediated trained immunity suggests that this pathway represents a potential therapeutic target to prevent ASCVD. Similar to findings reported for β-glucan-induced trained immunity [16], inhibiting the glycolytic activity of cells trained with oxLDL precluded the enrichment of H3K4me3 at pro-inflammatory cytokine promoters. Combined with our observations of H3K4me3 enrichment at the promoters of transcriptionally activated glycolytic genes, these findings further highlight the intimate bi-directional relationship between metabolic and epigenetic programming and the trained phenotype. While we demonstrate here the impact of oxLDL training on H3K4me3 at discrete promoters of immune genes and essential glycolytic genes, further study is needed to examine this histone modification epigenome-wide. In addition, investigation of other histone modifications such as H3K4 monomethylation, a characteristic feature of enhancers previously associated with trained immunity [28], could provide important mechanistic insight into the phenotype induced by oxLDL.

Accelerated atherosclerosis is the principal cause of mortality in patients with diabetes [20]. Our observation that glycolysis is upregulated in oxLDL-trained monocytes raises the intriguing possibility that increased glucose availability provides substrate for glycolytic metabolism, thereby amplifying the already heightened atherogenicity of trained macrophages [7]. In accordance with this hypothesis, GLUT1-mediated glucose metabolism was recently reported to drive a pro-inflammatory macrophage phenotype supported by glycolytic metabolism [29]. Our investigation of trained immunity under variable glucose concentrations demonstrates that high glucose exacerbates cytokine production in oxLDL-induced trained cells even further. In addition, we observed that glucose by itself can induce a trained phenotype in human primary macrophages. Previous studies have described an epigenetic memory of hyperglycemia in myeloid cells [30]; however, this phenomenon is yet to be explored thoroughly in the context of pro-inflammatory cytokine production. Therefore, our findings have broader implications for monocyte/macrophage function and phenotype in the disturbed metabolic environment of diabetes, which could translate to an increased risk for ASCVD [9]. Furthermore, we showed that metformin prevents oxLDL-induced trained immunity, suggesting that metformin could prevent atherosclerosis in patients for whom trained immunity is part of the pathophysiological process. Indeed, it has been speculated that metformin limits atherosclerosis by mechanisms independent of blood glucose lowering since it reduces cardiovascular disease compared with other antihyperglycemic drugs despite similar glycemic control. Inhibition of trained immunity might contribute to this beneficial cardiovascular effect of metformin.

In conclusion, we show distinguishable changes in the glucose metabolism of human primary monocytes mediated by brief exposure to a low concentration of oxLDL. Our cohort analyses revealed the importance of genetic variation in glycolytic regulators for the induction of trained immunity, and by targeting this metabolic pathway, we demonstrate the critical importance of glycolysis for the induction of a pro-inflammatory monocyte phenotype in oxLDL-trained macrophages. We propose that strategies interfering with glucose utilization specifically in the context of trained immunity may represent novel approaches to the treatment of vascular inflammation and atherosclerosis. However, further studies are necessary to strengthen the connection between our findings and human disease. For example, the use of recombinant high-density lipoprotein nanoparticles to deliver statins directly to macrophages in atherosclerotic lesions [31] could be similarly deployed to target specific aspects of glycolytic metabolism or epigenetic pathways implicated in trained immunity [3]. Further studies should expand on the role of epigenetic modifications in regulating key genes in atherogenic models of trained immunity, emphasizing the crucial connections between metabolites and chromatin-modifying reactions [3, 13, 32].

## Electronic supplementary material

ESM 1 (PDF 53 kb).

ESM 2 (PDF 50 kb).
