# Supplementary material for: Rewiring of glucose metabolism defines trained immunity induced by oxidized low-density lipoprotein
Source: J Mol Med (Berl). 2020 Apr 30;98(6):819–31. doi: 10.1007/s00109-020-01915-w (PMC7297856; doi:10.1007/s00109-020-01915-w)
Supplement: Supplementary file 1 — (PDF 53 kb). [file 109_2020_1915_MOESM1_ESM.pdf]

Table S1. Primers used for qRT-PCR analysis of mRNA expression and immunoprecipitated chromatin.

qRT-PCR primers for gene expression analysis		
**Gene**	**Forward(5'→3')**	**Reverse (5'→3')**
PFKFB3	ATTGCGGTTTTCGATGCCAC	GCCACAACTGTAGGGTCGT
PFKP	CGCCTACCTCAACGTGGTG	ACCTCCAGAACGAAGGTCC
HK2	TGCCACCAGACTAAACTAGACG	CCCGTGCCCACAATGAGAC
PKM1	CGAGCCTCAAGTCACTCCA	GTGAGCAGACCTGCCAGAC
PKM2	ATAACGCCTACATGGAAAAGTGT	TAAGCCCATCATCCACGTAGA
18s	GATGGGCGGCGGAAAATAG	GCGTGGATTCTGCATAATGGT
**qRT-PCR primers for analysis of immunoprecipitated chromatin**		
TNF promoter R1	CAGGCAGGTTCTCTTCCTCT	GCTTTCAGTGCTCATGGTGT
TNF promoter R2	AGAGGACCAGCTAAGAGGGA	AGCTTGTCAGGGGATGTGG
TNF promoter R3	GTGCTTGTTCCTCAGCCTCT	ATCACTCCAAAGTGCAGCAG
TNF promoter R4	TGTCTGGCACACAGAAGACA	CCCTGAGGTGTCTGGTTTTC
TNF promoter R5	TGATGGTAGGCAGAACTTGG	ACTAAGGCCTGTGCTGTTCC
IL6 promoter R1	TCGTGCATGACTTCAGCTTT	GCGCTAAGAAGCAGAACCAC
IL6 promoter R2	AGGGAGAGCCAGAACACAGA	GAGTTTCCTCTGACTCCATCG
PFKFB3 promoter R1	TCTTCTACTCGGGGCGATAA	GGAGCTGGACTGAAGTGGAC
PFKFB3 promoter R2	TCAAGGTTCCCAGTCTTTGG	CACCTCGTCCTTGTCCACTT
PFKFB3 promoter R3	ACTGCGTCTCTGCTCTCCTC	GCTGCTGTTATCCCCTCGT
HK2 promoter R1	GAGCTCAATTCTGTGTGGAGT	ACTTCTTGAGAACTATGTACCCTT
PFKP promoter R1	CGAAGGCGATGGGGTGAC	CATCGCTTCGCCACCTTTC
