# Supplementary material for: Rewiring of glucose metabolism defines trained immunity induced by oxidized low-density lipoprotein
Source: J Mol Med (Berl). 2020 Apr 30;98(6):819–31. doi: 10.1007/s00109-020-01915-w (PMC7297856; doi:10.1007/s00109-020-01915-w)
Supplement: Supplementary file 2 — (PDF 50 kb). [file 109_2020_1915_MOESM2_ESM.pdf]

Table S2. Baseline characteristics of the 11 participant of the metformin trial.

**Parameter**	**Mean (SD)**
Sex	All males
Age (y)	21.8 (1.9)
Height (m)	1.83 (0.07)
Weight (kg)	71.7 (8.4)
BMI (kg/m2)	21.4 (1.7)
Creatinine (µmol/l)	77.7 (7.0)
Total cholesterol (mmol/l)	4.3 (0.6)
Triglycerides (mmol/l)	1.2 (0.6)
HDL-cholesterol (mmol/l)	1.2 (0.4)
LDL-cholesterol (mmol/l)	2.4 (0.7)
HbA1c (mmol/mol)	32 (2)
